# Analysis of *her1* and *her7* Mutants Reveals a Spatio Temporal Separation of the Somite Clock Module

**DOI:** 10.1371/journal.pone.0039073

**Published:** 2012-06-18

**Authors:** Suma Choorapoikayil, Bernd Willems, Peter Ströhle, Martin Gajewski

**Affiliations:** 1 Institute for Genetics, University to Cologne, Koeln, Germany; 2 Department of Biological Sciences and National University of Singapore Centre for BioImaging Sciences, National University of Singapore, Singapore, Singapore; Texas A&M University, United States of America

## Abstract

Somitogenesis is controlled by a genetic network consisting of an oscillator (clock) and a gradient (wavefront). The “*hairy* and *Enhancer of Split*”- related (*her*) genes act downstream of the Delta/Notch (D/N) signaling pathway, and are crucial components of the segmentation clock. Due to genome duplication events, the zebrafish genome, possesses two gene copies of the mouse *Hes7* homologue: *her1* and *her7*. To better understand the functional consequences of this gene duplication, and to determine possible independent roles for these two genes during segmentation, two zebrafish mutants *her1^hu2124^* and *her7^hu2526^* were analyzed. In the course of embryonic development, *her1^hu2124^* mutants exhibit disruption of the three anterior-most somite borders, whereas *her7^hu2526^* mutants display somite border defects restricted to somites 8 (+/−3) to 17 (+/−3) along the anterior-posterior axis. Analysis of the molecular defects in *her1^hu2124^* mutants reveals a *her1* auto regulatory feedback loop during early somitogenesis that is crucial for correct patterning and independent of *her7* oscillation. This feedback loop appears to be restricted to early segmentation, as cyclic *her1* expression is restored in *her1^hu2124^* embryos at later stages of development. Moreover, only the anterior *deltaC* expression pattern is disrupted in the presomitic mesoderm of *her1^hu2124^* mutants, while the posterior expression pattern of *deltaC* remains unaltered. Together, this data indicates the existence of an independent and genetically separable anterior and posterior *deltaC* clock modules in the presomitic mesdorm (PSM).

## Introduction

Somitogenesis is an essential and complex process during early vertebrate development. As the body axis elongates, transient metameric structures, called somites, bud off from the PSM at the tail bud adjacent to both sides of the notochord. This complex process requires the carefully coordinated activation and inhibition of gene transcription and is controlled by a molecular oscillator [1]–[4]. Extensive studies have been carried out to elucidate the mechanisms that control cyclic gene expression, revealing important roles for signaling pathways such as D/N-, Wnt- and FGF-signaling. However, the genetic network and interplay between these pathways is not fully understood yet. Typically, loss of function of one component in this network does not lead to breakdown of the whole process. Instead, only partial somitic defects occur at distinct positions along the body axis. Thus, it seems likely that the system possesses the ability to compensate for the loss of individual signal inputs found in loss of function situations [5]–[8]. Alternatively, it suggests that during embryonic development multiple mechanisms exist to control segmentation over time.

The process of somitogenesis commences when the first anlagen of the somites are generated and involves three steps that are essential for somite formation. First, the unsegmented PSM is pre-patterned, followed by the establishment of rostro-caudal (r/c) polarity and finally by the formation of somitic borders [9], [10]. However, it remains elucidated whether these three steps are functionally linked or are driven by independent mechanisms. One of the major pathways involved in the process of pre patterning is the D/N-signaling pathway. The components of the D/N pathway, together with their target genes from the *hairy* and *enhancer of Split (hes)* family, constitute a genetic feed-back loop [11], [12] which ultimately results in cyclic gene expression. Morpholino oligonucleotide (MO) mediated knock down studies in zebrafish have shown that loss of Her function disrupts the cyclic expression of D/N components, suggesting an important role for Her transcription factors in the D/N-mediated oscillation mechanism [13], [14].

*Her* genes encode basic Helix-Loop-Helix (bHLH) transcription factors, which act in a protein complex with the co-repressor Groucho [15]. Due to a gene duplication, zebrafish possess two homologues of murine *Hes7* [16], annotated as *her1* and *her7*. Both genes have been reported to play important and separate roles during pre patterning of the unsegmented PSM. MO mediated knock down studies indicate an essential requirement for *her1* in the formation of the first three somites [14], whereas *her7* was shown to play a role in segmentation posterior to the ninth somite [6]. Moreover, loss of function of both *her* genes, either in the *b567* mutant or through MO mediated knock down [14], results in disruption of all somites. These findings suggest non-redundant roles or temporally separate roles for both *her* genes during specific stages of segmentation.

In this study, we present novel zebrafish *her1* and *her7* mutants, and analyse the role of both *her* genes in pre-patterning of the PSM during early embryonic development. Furthermore, we analyse PSM pre-patterning in double-mutant fish lacking both DeltaC and Her1 function. Expression analysis of the clock genes in double-mutant embryos revealed a critical role for Her7 dependent posterior PSM oscillations in the synchronization of gene expression in adjacent cells. In contrast, we found that Her1 drives the pre-patterning of the first three somites in the anterior PSM. Together, our study demonstrates distinct spatio-temporal requirements for *her1* and *her7* during somite formation.

## Results and Discussion

### Characterisation of the *her1* and *her7* Mutant Alleles

ENU-induced point mutations were identified in the *her1* and *her7* genes by 5′-end sequencing of the relevant genomic DNA derived coding sequences amplified from mutagenized fish.

**Figure 1:**
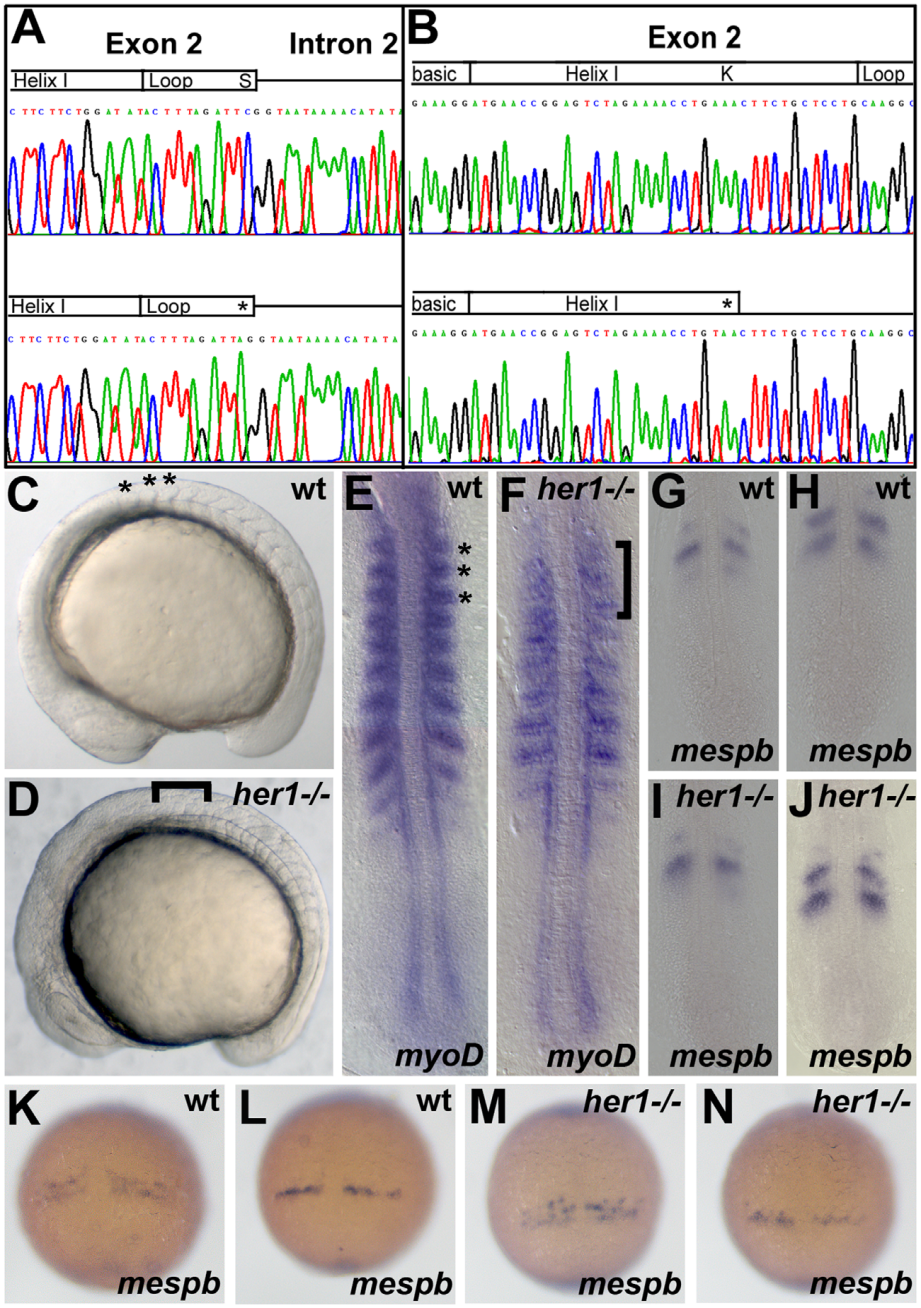
*Her1* mutants exhibit defects in somitogenesis. Electropherogram of *her1* (A) and *her7* (B) amplicons in wild type (top), homozygous *her1^hu2124^* (bottom, A) and *her7^hu2526^* (bottom, B) mutant fish. Schematics above the sequences depict the exon- and intron-organization and the protein domains encoded by the exons. Point mutations are indicated by asterisks. (C, D) Brightfield pictures of wild type and *her1* mutant embryos, lateral view, anterior to left. Compared to wild type embryos (C, asterisks), the first 3 somitic borders in the *her1^hu2124^* mutant appear diffuse and partly disrupted (D, bracket). In situ analysis of *myoD* expression in wild types indicates characteristic half-segmental expression within the somites (E, asterisks indicate somites 1–3). *myoD* expression is diffuse in the first 3 somites of *her1^hu2124^* embryos (F, bracket). (G, H) and (K, L) show half-segmental respectively r/c polarity wild type expression pattern of *mespb* at 10–12 somite stage and between 90% epiboly and bud stage, respectively. (I, J) and (M, N) represents *mespb* expression in the *her1^hu2124^* mutant at 10–12 somite stage and between 90% epiboly and bud stage, respectively. Expression of *mespb* is disturbed in the *her1^hu2124^* mutant between 90% epiboly and bud stage (M, N), when the anlagen of the first somites are pre-patterned. Compared to one or two stripes in the wild-type (K, L), *mespb* is expressed in a salt and pattern (M, N). *mespb* expression is unperturbed at 10–12 somite stage in *her1^hu2124^* mutants (I, J). Dorsal views, anterior to top.

One allele with a single base pair transition was identified for each gene. The *her1^hu2124^* allele (acc no X97329) contains a C>A transition at position 185, resulting in a premature stop codon (TCG(S)>TAG/(stop)). The *her7^hu2526^* allele (acc no AF240772, [17] contains an A>T transition at position 208, also resulting in a premature stop codon (AAA(K)>TAA/(stop) (Fig. 1A,B). In both mutants the stop-codon is located upstream of the basic domain. *her1^hu2124^* is truncated within the loop located at the end of exon 2, and *her7^hu2526^* is truncated within HelixI of the HLH-domain located in exon 2. Thus, both mutant proteins lack a full HLH-domain, and are hypothesized to lack dimerization function.

### Her1 is required for Patterning the Anterior-most Somites

Previous studies have demonstrated that *her1*-morphant embryos show a spectrum of phenotypes ranging from mild morphological defects in the anterior 1 to 3 somites, to more severe defects observed along the entire axis [6], [13], [14]. This variability in phenotype may be attributed to incomplete knock down using MO, and therefore can make it difficult to determine precisely the requirement for Her1 during segmentation. Therefore, to better understand the function of Her1 during early somitogenesis, we compared segmentation events between *her1^hu2124^* homozygous mutant embryos and wild type siblings. Whereas wild type siblings showed normal somite formation (Fig. 1C), *her1^hu2124^* homozygous mutant embryos exhibit defects in the borders of the first (anterior) somites (Fig. 1D). Consistently, analysis of *myogenic differentiation 1* expression (*myoD*, [18]) reveals a diffuse pattern within the misshapen somites of *her1^hu2124^* mutant embryos when compared to wild type embryos or to more posterior somites in the mutant (Fig. 1E, F).

**Figure 2:**
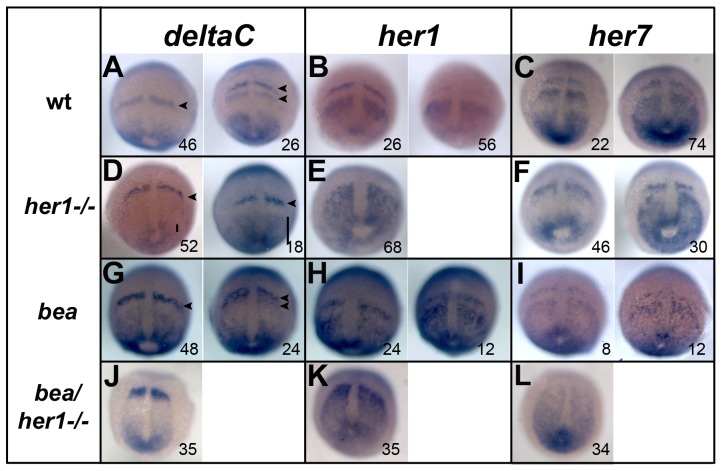
Expression analysis of segmentation genes in *her1^ hu212^* and *bea^tm98^* mutants. In situ hybridisation analysis of segmentation clock genes *deltaC, her1*, and *her*7 in wild type (A–C) *her1^hu2124^* mutants (D–F), *bea^ tm98^* mutants (G–I) and *her1^hu2124^*/*bea^ tm98^* double mutants (J–L) at 90% epiboly. Cyclic *deltaC* expression is disrupted in the anterior PSM of *her1^hu2124^* mutants. Instead of one or two expression stripes as in the wild type (A, arrowheads) only one stripe of expression is observed (D, arrowhead). Expression domains in the posterior PSM display different sizes indicating unperturbed oscillation of *deltaC* in the tail bud of *her1^hu2124^* mutants (D, bars). Cyclic expression of *her1* is fully disrupted in the *her1^hu2124^* mutant (E) when compared to wild type (B), whereas *her7* expression remains oscillatory (compare C and F). Cyclic expression of all three genes is observed in *bea^tm98^* although some slight initial perturbation is observed (G-I). In *her1^hu2124^*/*bea^tm98^* double mutants, all three clock genes show fully disrupted expression patterns at 90% epiboly. Dorsal views, anterior to the top, number in each panel indicate cycling phases.

**Figure 3:**
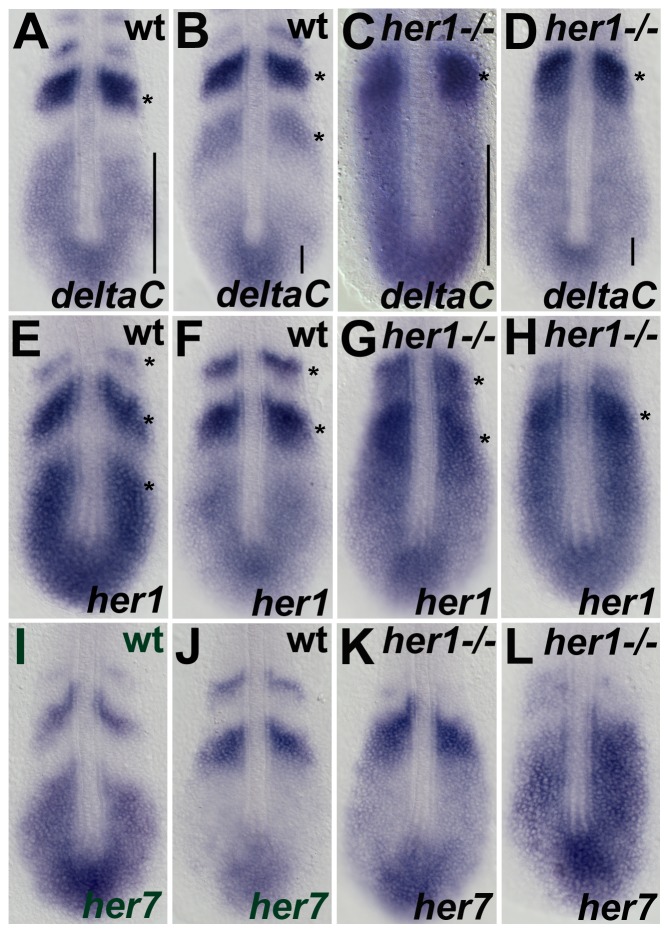
Expression analysis of the segmentation clock genes at 10–12 somite stage in *her1^hu2124^* mutants. In situ hybridisation analysis of the segmentation clock genes *deltaC*, *her1*, and *her7*, in wild type embryos (A,B,E,F,I,J) *her1^hu2124^* mutant (C,D,G,H,K,L) at the 10–12 somite stage. Two significantly different patterns are shown for each gene to indicate oscillatory expression. Expression of *deltaC* in *her1^hu2124^* mutants at this developmental stage is identical to the 90% epiboly (see Fig. 2D), cyclic in the posterior PSM and disrupted expression in the anterior PSM (C, D) compared to wild type (A, B). Expression of *her1* and *her7* oscillates in the *her1^hu2124^* mutant but on average one expression stripe is lacking (see asterisks in G, H and K, L, respectively) compared to the respective wild type expression domains (asterisks in E, F and I, J). Further, the patterns in the PSM of mutants appear stretched towards the anterior compared to wild type (see bars in A-D) suggesting that one expression wave is lacking. Dorsal view, anterior to the top.

To determine the requirement for Her1 in establishing r/c polarity, the expression pattern of *mesoderm posterior* (*mesp*) [19] was compared in wild type and *her1^hu2124^* mutant embryos. *mespb* expression in *her1^hu2124^* mutant embryos was disrupted during the pre-patterning of somites 1 to 3. While wild type embryos display a stripe expression pattern of *mespb* (Fig. 1K, L), a “salt and pepper”-like expression pattern was observed in the *her1^hu2124^* mutant (Fig. 1M, N). During later stages of segmentation, when border formation is unaffected in the *her1^hu2124^* mutant, wild type-like expression of *mesp* is restored (Fig. 1G–J). This indicates that the maintenance of r/c polarity in the anterior-most somites is regulated through Her1 activity. To understand the relationship between the morphological somite defects observed in the *her1^hu2124^* mutant and the molecular oscillation clock, the expression patterns of *deltaC*, *her1* and *her7* were examined between 90% eiboly and bud stage, when the first 3 somites are pre-patterned (Fig. 2). While wild type embryos display cyclic *deltaC* expression (Fig. 2A), *her1^hu2124^* mutants exhibit disruption of the cyclic *deltaC* expression in the anterior PSM (Fig. 2D). Only one *deltaC* expression domain is detectable in the Her1 loss of function situation. Importantly, oscillating *deltaC* expression in the posterior PSM was detected in both wild type (Fig. 2A) and *her1^hu2124^* mutant embryos (Fig. 2D), indicating that cyclic *deltaC* expression in the posterior PSM is independent of Her1 function. To further confirm both Her1-dependent and -independent *deltaC* oscillations, *deltaC* expression was analyzed at the 10–12 somite stage, when somite border defects are no longer observed in *her1^ hu2124^* mutants (Fig. 3). At this stage, *her1^ hu2124^* mutants express only a single stripe of *deltaC* in the anterior PSM, in contrast to the 1–2 stripes of expression observed in wild type embryos, indicating that cyclic *deltaC* expression in the anterior PSM is indeed dependent on Her1 activity (Fig. 2A, D). In contrast, different phases of oscillation in the posterior PSM were detected in both, wild type embryos (Fig. 2A) and in *her1^ hu2124^* mutant embryos (Fig. 2D), indicating that *deltaC* expression oscillates in the absence of functional Her1 in the posterior PSM. Thus, the absence of Her1 leads to impaired *deltaC* expression in the anterior PSM, whereas cyclic gene expression in the posterior PSM is not affected. These findings support the conclusion that cyclic *deltaC* expression in the posterior part of the PSM occurs independent of Her1. Furthermore, our investigation suggests that two *deltaC* clock modules exist, in which the posterior and anterior *deltaC* expression waves are driven separately. Although loss of Her1 activity results in disruption to both anterior *deltaC* expression and formation of anterior somite borders, later during segmentation these somite borders are restored while *deltaC* expression remains disrupted in the *her1^ hu2124^* mutant. It is therefore unlikely that the morphological somite defects in *her1^ hu2124^* mutant embryos are caused by disrupted *deltaC* expression.

Next, the expression pattern of *her* genes in the *her1^hu2124^* mutant was analyzed. Cyclic expression of *her1* is disrupted in *her1^hu2124^* homozygous mutants between 90% epiboly and bud stage (Fig. 2B, E). In contrast, oscillation of *her7* is not affected at this stage in the *her1^hu2124^* mutant (Fig. 2C, F), suggesting that Her1 negatively regulates its own expression, but is not required for *her7* expression during early segmentation. Interestingly, during later segmentation stages oscillating *her1* expression patterns are observed (Fig. 2E–H), demonstrating that *her1* resumes oscillation over the course of development, even in the absence of Her1. However, the domain of cyclic expression of both *her1* and *her7* in the posterior PSM appears expanded anteriorly, and with a simultaneous lack of an expression wave (Fig. 3E–L). Nevertheless, defects in somite formation are not observed in later stages, indicating that altered *her1* and *her7* expression does not affect somite boundary formation. Thus, Her1 acts in a temporally restricted manner and contributes to the segmentation clock independent of the DeltaC-Her7 feedback loop during early development.

### *her7* and *deltaC* Oscillation are Regulated Through Her1 During Early Development

**Figure 4:**
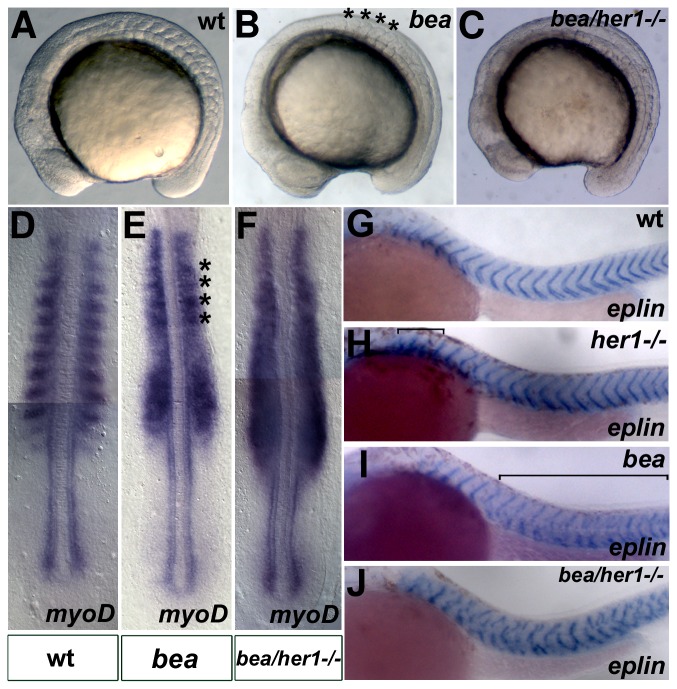
Analysis of the *her1^hu2124^*/*bea^ tm98^* double mutant phenotype. Brightfield images of wild type, *bea^tm98^* and *her1^hu2124^*/*bea^tm98^* mutant embryos at the 10–12 somite stage, lateral views, anterior to left. Compared to the wild type embryo (A), the somite borders posterior of the 4^th^ somite are disrupted in the *bea^tm98^* mutant (B, asterisks indicate correctly formed somites). All somitic borders are disrupted in the *her1^hu2124^*/*bea^tm98^* double mutant (C). In situ hybridisation analysis of *myoD* expression at 10–12 somites (D–F), dorsal views, anterior to top. In line with the morphological phenotypes, half segmental *myoD* expression is disrupted posterior to the 4^th^ somite in *bea^tm98^* (E, asterisks mark residual expression in somites 1–4) and along the entire body axis in *her1^hu2124^*/*bea^tm98^* double mutants (F) compared to wild type (D). In situ analysis of *eplin* expression at prim 6 stage (G–J) lateral views, anterior to left. *eplin* is expressed in v-shape at the somite borders in the wild-type (G). Disturbed *eplin* expression is observed in the first somites of the *her1^hu2124^* mutant (H, bracket), posterior to the somite 4 in the *bea^tm98^* mutant (I, bracket) and in all somites in the double mutant situation (J). (A–F) 10–12 somite stage, (G–J) prim 6 stage.

*bea/deltaC* mutant embryos exhibit segmentation defects along their antero-posterior axis, beginning between the third and fifth somite. In addition to these morphological defects, expression analysis revealed that expression of segmentation clock genes is perturbed (Fig. 4A, B; [20], [21]). Examination of *her1^ hu2124^* mutant embryos revealed a complementary pattern of somite disruption, whereby only the first three somite borders are disrupted (Fig. 1D). To better understand the relationship between DeltaC and Her1, homozygous double mutant embryos for *her1* and *deltaC* were created and somite border defects were analyzed and compared between double mutants, single mutants and wild type embryos (Fig. 4A–C). *her1^hu2124^*/*bea^tm98^* homozygous double mutants show disruption of somitic borders along the entire axis (Fig. 4C). In addition, half segmental (Fig 4D) expression of *myoD* is disrupted in all somites (Fig. 4F), compared with the restricted anterior perturbation in *her1^hu2124^* mutants (Fig. 1F) and the defects observed in *bea^ tm98^* mutants starting from somites three to five (Fig. 4E). The same segmentation defect was observed by analyzing expression of a segment border marker. In wild type embryos at prim-6 stage *eplin* is expressed along the segment borders in a characteristic v-shape (Fig. 4G, [22]. This pattern is disrupted in the three anterior-most somites in *her1^hu2124^* mutants (Fig. 4H). In *bea^ tm98^* mutants expression of *eplin* in all somites but the first three or four are disrupted (Fig. 4I). Double *her1^hu2124^*/*bea^ tm98^* mutants display disrupted *eplin* expression along the whole axis (Fig. 4J).

To investigate the influence of the loss of both Her1 and DeltaC on the segmentation clock, the expression of *deltaC*, *her1* and *her7* was examined in embryos between 90% epiboly and bud stage and compared to the expression patterns observed in single mutants and wild type embryos. Analysis of *bea^ tm98^* mutants revealed that the expression pattern of all three genes oscillates normally prior to the three somite stage, although expression is slightly diffuse compared to the wild type embryos (Fig. 2G, H, I, respectively, [20], [21]. In *her1^hu2124^* mutants, as described above, expression of *her1* is perturbed (Fig. 2E) and *deltaC* oscillation is only disrupted in the anterior PSM (Fig. 2D), whereas *her7* expression appears cyclic (Fig. 2F). In contrast, cyclic *her7* expression in *her1^hu2124^*/*bea^ tm98^* double mutant embryos is completely disrupted, with *her7* expressed in a gradient with declining expression from posterior to anterior (Fig. 2L). In addition, posterior *deltaC* oscillation is disrupted in the double mutant (Fig. 2J) when compared with the *her1^hu2124^* mutant (Fig. 2D). Instead of two different expression phases, which were observed in the posterior PSM in the *her1^ hu2124^* mutant embryos (Fig. 2D), an invariant posterior expression pattern of *deltaC* was observed in *her1^ hu2124^*/*bea^ tm98^* double mutants (Fig. 2J). Thus, cyclic expression of all three analyzed clock genes is completely disrupted in *her1^ hu2124^*/*bea^ tm98^* double mutant embryos from the time point of initiation of segmentation. This indicates that cyclic *her7* expression and posterior *deltaC* oscillation are regulated in a combinatorial manner through both a Her1 auto regulatory feedback loop and a D/N signaling module.

### Analysis of Segmentation Clock Genes in *her7^hu2526^* Mutant Embryos

**Figure 5:**
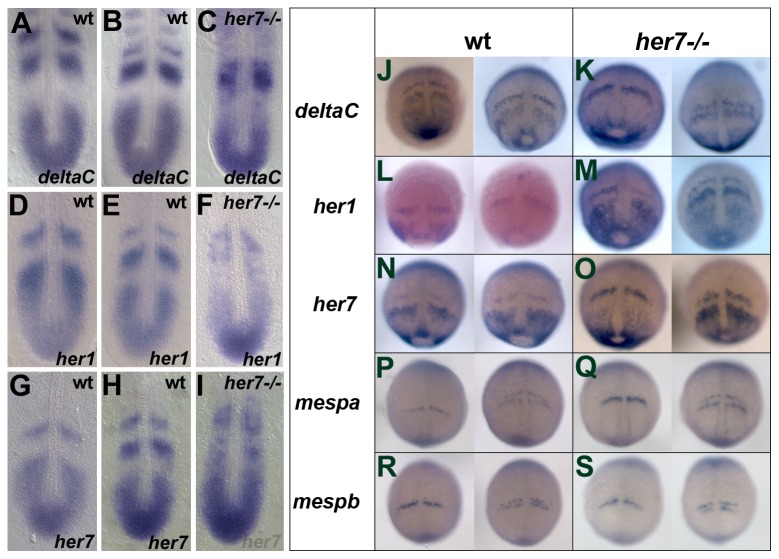
Expression analysis of segmentation genes in *her7^hu2526^* mutant embryos. In situ hybridisation analysis of *deltaC*, *her1* and *her7* in wild type (A, B and D, E and G, H, respectively) and *her7^hu2526^* mutants (C, F, I, respectively) at 10–12 somite stage and between 90% epiboly and bud stage (J, L, N for wild type expression patterns and K, M, O for respective expression patterns in the mutant embryos). Expression patterns of *deltaC*, *her1* and *her7* at 10–12 somite stage are disrupted in the mutant appear unperturbed between 90% epiboly and bud stage. Expression patterns of *mespa* and *mespb* are not affected in the *her7^hu2526^* mutant between 90% epiboly and bud stage (Q and S, respectively) and similar to the wild type (P and R, respectively).

In light of the phenotypic variability observed in *her1* morphants, we re-analyzed the expression of the clock genes *her1*, *her7* and *deltaC* during somitogenesis in *her7^hu2526^* mutants. Cyclic expression of *deltaC* is disrupted in *her7^hu2526^* mutant embryos (Fig. 5 A–C), similar to those phenotypes observed in *her7* morphants, or in *D/N* mutants [6], [13], [23] at the 10–12 somite stage. Expression of *her1* and *her7* is disrupted in *her7^hu2526^* homozygous mutants in a similar manner to that observed in the *her7* morphant (Fig. 5D–I). Thus, *her7* morphants and *her7^hu2526^* mutants show similar disruption of the segmentation clock genes at the 10–12 somite stage. Furthermore, we found that expression of all examined clock genes is unperturbed during early somitogenesis (Fig. 5J–O). D/N mutants, such as *bea*, *des*, *aei* or *mib,* or MO mediated knock down of *deltaC*, *notch1a*, *deltaD* and *E3 ligase,* display somitic border defects from the 3^rd^, 7th, 8th and 9th somite onwards, respectively. In line with the observed border defects cyclic gene expression of *deltaC*, *her1* and *her7* are disrupted [5], [6], [21], [24]. In a similar fashion, cyclic gene expression of *deltaC*, *her1* and *her7* in *her7^hu2526^* mutants are disrupted in conjunction with somitic border malformation.

### Her7 Plays an Essential Role During Pre-patterning

**Figure 6:**
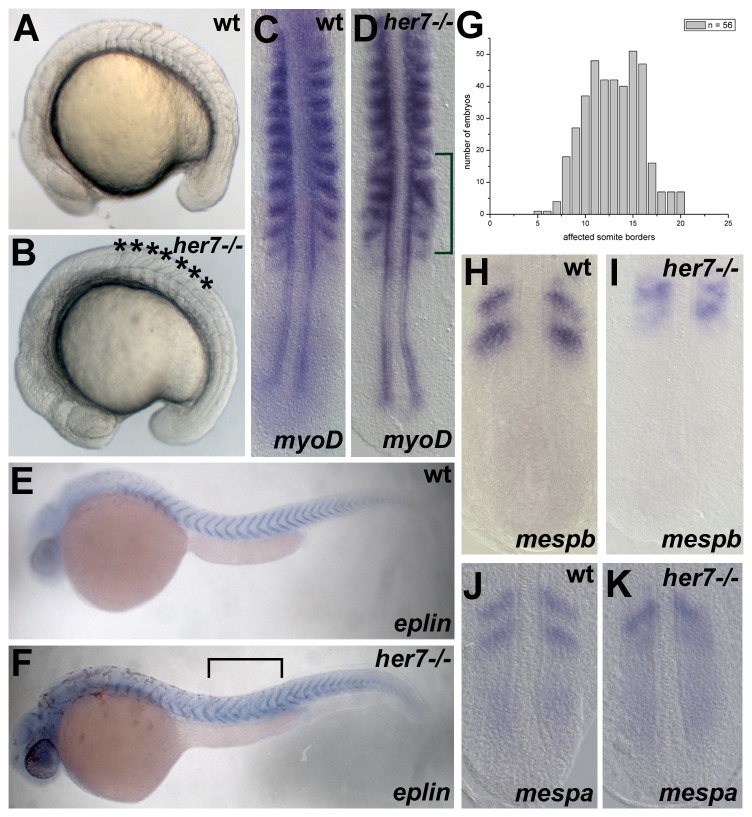
The role of Her7 during pre-pattering. Brightfield images of wild type and *her7* mutant embryos at 16–18 somites (A, B). Compared to the wild type embryo (A), somite borders posterior to the 8^th^ somite are disrupted in the *her7^hu2526^* mutant (B, bracket). In situ hybridisation analysis of *myoD* expression (C, D), *eplin* (E, F), *mespb* (H, I) and *mespb* (J, K) in wild type and *her7* mutants. Compared to half-segmental *myoD* expression in the wild type (C), *myoD* expression is disrupted posterior to the 8^th^ somite in *her7^hu2526^* mutants at 10–12 somites (D, bracket). In addition to the ALD at somite 8, *her7^hu2526^* mutant larvae show a PLD at around somite 17 (F, bracket indicates area of defect). *eplin* expression posterior to the PLD appears V-shaped as in wild-type at prim 6 stage (compare E, F). (G) graph plotting the number of *her7^hu2526^* embryos exhibiting defective somites (n = 56) as a function of their respective position along the a/p-axis of the animal. The obtained formula for the defect in the *her7^hu2526^* mutant is 8 (+/−3)−17 (+/−3) indicating that in some rare cases the defects seem to appear at both the ALD and the PLD with a slight variability. *mespb* expression in the wild type and the *her7^hu2526^* mutant are shown in (H) and (I), respectively. (J) and (K) *mespa* expression in the wild type and *her7^hu2526^* mutant, respectively. Expression of both genes is disrupted in the *her7^hu2526^* mutant at 10–12 somite stage. (A, B, E, F) lateral view, anterior to the left; (C, D, H-K) dorsal view, anterior to the top.

To determine the temporal onset of somite defects in *her7^hu2526^* mutant embryos, *myoD* expression was examined at 12–14 somite stage. The anterior limit of somitic boundary defects (ALD) in the *her7^hu2526^* mutant was observed around the level of the 8^th^ somite (Fig. 6A, B). The *myoD* expression pattern was disturbed at the same axial level (Fig. 6C, D [6]). To examine the posterior extent of somitic defects, *eplin* expression was analysed in the mutants after completion of somitogenesis, permitting visualization of the somite borders. In *her7^hu2526^* mutant embryos *eplin* expression is disrupted with high penetrance between somite 8(+/−3) to somite 17(+/−3) (n = 56, Fig. 6F and graph in Fig. 6G). Somitic borders posterior to this region appear unaffected indicating that a posterior limit of defects (PLD) exists in upon Her7 loss-of-function. In line with this finding, disrupted *mesp* expression was observed during, but not prior to, this time interval (Fig. 6H–K,). Thus, Her7 has a non-redundant role in somite border formation between the ∼8^th^ and ∼17^th^ somite.

In summary, molecular and morphological analysis of *her1* and *her7* mutants indicate a non-redundant requirement for both these genes in the correct segmentation of distinct somite regions in the zebrafish. Our data resolves previous seemingly contradictory data arising from *her1* morphant analysis [23] and *in vitro* studies with *her1* promoter constructs [25]. Observations in the latter study strongly supported a *her1* negative auto regulation mechanism; however *in vivo* analysis of the *her1* morphant did not provide any supporting evidence for this conclusion. Our *her1^hu2124^* mutant analysis now suggests that the regulatory requirement of Her1 decreases during the course of segmentation. During early somitogenesis, Her1 activity constitutes a negative auto regulatory feedback loop, in agreement with the findings of Kawamura et al., 2005 [25], while later during development the auto regulatory potential of Her1 is considerably reduced or absent, as suggested by the residual expression in the inter stripe regions of *her1^hu2124^* mutants. Furthermore, Her1 does not negatively feed back on *her7* in a direct manner, at either early or late somitogenesis, as our own study previously has suggested [23]. However, Her1 is required to regulate the rhythm of *her* gene oscillation, as shown by the altered expression patterns that suggest an increase in wavelength towards the anterior. This effect is most probably caused indirectly by loss of the repressive activity of Her1 on *delta* gene expression. Nevertheless, the increase in wavelength towards the anterior is not associated with changes in somite size (data not shown). Furthermore, analysis of clock genes in *her1^hu2124^*/*bea^tm98^* double mutants revealed that cyclic *her7* expression and posterior *deltaC* oscillation in the PSM are governed by a Her1 auto regulatory feedback loop. Morphologically, *her1^hu2124^*/*bea^tm98^* double mutant exhibit a cumulative phenotype, strongly supporting distinct roles for Her1 and DeltaC during somitogenesis. Future studies should seek to identify the D/N independent Her1 targets that control anterior somite formation. Moreover, *her7^hu2526^* mutant analysis confirmed the role previously suggested for *her7* in somitogenesis during the 1–12 somite stage. In addition, the observation of a PLD in the *her7^hu2526^* mutant further suggests a temporally restricted role for Her7 during somitogenesis.

In summary, the comparison between single *her1^hu2124^* and *her7^hu2526^* mutants and *her1^hu2124^*/*bea^ tm98^* double mutants suggests independent roles for both *her* genes in regulation of distinct phases of the segmentation clock. There subsequently remains an open question about the direct downstream targets of DeltaC, which together with Her1 are able to initiate cyclic *her7* expression.

## Materials and Methods

### Ethic Statement

Adult zebrafish were handled according to relevant national and international guidelines and was approved by the German environment and customer protection office Cologne (§ 11 Abs. 1 No. 1 for animal protection law (BGBL.I.S. 1005–1120). Only embryos up to 32 hpf were used for these experiments, which do not require approval of the animal experiments committee according to national and European law.

### Genotyping and Used Mutant Fish

Fish were maintained at 28.5°C on a 14-h light/10-h dark cycle. Embryos were collected by natural spawning and staged according to Kimmel et al., 1995 [26].

*her1* and *her7* heterozygous mutants were identified by screening the ENU-mutagenised Tilling Library at the Hubrecht Institute, Utrecht. To identify *her1* and *her7* homozygous carriers, the 5′ end of the relevant gene was amplified from genomic DNA from fin clips and analyzed by sequencing. The *her1^hu2124^* or *her7^hu2526^* alleles, respectively, were genotyped by PCR using the following primers: her1F 5′-GAG AAG AAA CGG AGA GAC CGG-3′ and her1R 5′- CTT TAC ATA CGT GTA GAC AGG-3′; her7F 5′-GAT GAA AAT CCT GGC ACA GAC T-3′ and her7R 5′-TCT GAA TGC AGC TCT GCT CG-3′. The amplicons were purified using AcroPrep^TM^96 plates (PALL) and sequenced.

The *bea^tm98^* mutant was used in this study [27].

### In situ Hybridisation

Riboprobes for *her1*, *her7*, *deltaC* and *myoD* were generated as described [7], [23]. *mespa* and *mespb* amplicons were generated with mesp-a T3 fw 5′-AAT TAA CCC TCA CTA AAG GGT GCT GTA TCA GAT GC-3′, mesp-a T7 rv 5′-TAA TAC GAC TCA CTA TAG GGT CAC CTT GAA CTG GA-3′ and mesp-b T3 fw 5′-AAT TAA CCC TCA CTA AAG GGA CGC TAG TGA GAA GG-3′, mesp-b T7 rv 5′- TAA TAC GAC TCA CTA TAG GGG CCC ACA CTG TTG AC-3′, respectively. As a somitic boundary marker the cb1045 (*eplin*) probe was used as described [28].

Automated in situ hybridization was carried out following the protocol of Leve et al., 2001 [17] using a programmable liquid handling system (InsituPro, Intavis) described by Plickert et al., 1997 with a hybridization temperature of 65°C. Digoxygenin-labeled RNA probes were prepared using RNA labeling kits (Roche). Staining was performed with BM purple (Roche). Whole-mount embryos were observed under a stereomicroscope (Leica) and digitally photographed with Leica DFC 480. Flat mounted embryos were analyzed with an Axioplan2 microscope connected to an Axiocam system (Zeiss).
